# Developments in Diagnosis and Antileishmanial Drugs

**DOI:** 10.1155/2012/626838

**Published:** 2012-10-18

**Authors:** Prachi Bhargava, Rajni Singh

**Affiliations:** ^1^Amity Institute of Biotechnology, Amity University Uttar Pradesh, Sector 125, Noida 201303, India; ^2^Amity Institute of Microbial Biotechnology, Amity University Uttar Pradesh, Sector 125, Noida 201303, India

## Abstract

Leishmaniasis ranks the third in disease burden in disability-adjusted life years caused by neglected tropical diseases and is the second cause of parasite-related deaths after malaria; but for a variety of reasons, it is not receiving the attention that would be justified seeing its importance. Leishmaniasis is a diverse group of clinical syndromes caused by protozoan parasites of the genus *Leishmania*. It is estimated that 350 million people are at risk in 88 countries, with a global incidence of 1–1.5 million cases of cutaneous and 500,000 cases of visceral leishmaniasis. Improvements in diagnostic methods for early case detection and latest combitorial chemotherapeutic methods have given a new hope for combating this deadly disease. The cell biology of *Leishmania* and mammalian cells differs considerably and this distinctness extends to the biochemical level. This provides the promise that many of the parasite's proteins should be sufficiently different from hosts and can be successfully exploited as drug targets. This paper gives a brief overview of recent developments in the diagnosis and approaches in antileishmanial drug discovery and development.

## 1. Introduction

Protozoan parasitic diseases remain a major concerned public health problem, especially in tropical regions. The major death toll is due to malaria, African and American trypanosomiasis, and leishmaniasis, whose high mortality rates in underdeveloped developing countries are associated to poor hygienic conditions and lack of efficient prophylactic measures [1]. 

For many years, the public health impacts of the parasitic diseases have been grossly underestimated, mainly due to lack of awareness of its serious impact on health. Protozoan parasites of the genus *Leishmania* cause severe diseases that threaten human beings, both for the high death rates involved and the economic loss resulting from morbidity, primarily in the tropical and subtropical areas [2]. It ranks the second only to malaria, and the control of leishmaniasis remains a serious problem with ever increasing cases worldwide. It has become a major focus of concern and a serious third world problem affecting the poorer sections of the society [3]. 

Leishmaniasis is included in the list of neglected tropical diseases (NTDs) [4] and has a strong link to poverty [5]. The disease has been reported in 88 countries in five continents—Africa, Asia, Europe, North America, and South America out of the seven continents (22 in the new world and 66 in the old world) [6], out of which 16 are developed countries, 72 are developing, and 13 of them are among the least developed [7]. Approximately, 350 million individuals are at risk of leishmaniasis and 20 million people are infected worldwide, and an estimated 2.0 million new cases occur each year [8] with an incidence of 1.5 million cases per annum of the disfiguring cutaneous leishmaniasis (CL) and 0.5 million cases per annum of the potentially fatal visceral leishmaniasis (VL) [9, 10]. However, with increasing travel to and from endemic regions, there is an increase in the number of patients suffering from leishmaniasis [10–12]. The importance of this parasitic disease is further stressed out by the rise of *Leishmania*/HIV coinfection in many parts of the world including European countries such as Spain, Italy, France, and Portugal where up to 9% of the AIDS patients suffer from fatal visceral leishmaniasis [13]. However, due to underreporting and misdiagnosis, the number of actual cases is expected to be higher. 

No effective vaccines are available against *Leishmania* infections as yet and the treatment relies solely on chemotherapy, with pentavalent antimonials as first-line drugs and amphotericin B and pentamidine as second-line agents [10, 11]. Miltefosine is the first recognized oral treatment for leishmaniasis, but resistance to miltefosine may emerge easily during treatment due to single point mutation [14]. There is a pressing need for the identification of novel drug targets, virulence factors, and development of vaccines to expand our understanding of the prevention and treatment of leishmaniasis.

### 1.1. Epidemiology: Impact and Geographical Distribution

Leishmaniases cause considerable morbidity and mortality and are a typical example of an anthropozoonosis. The majority of infections are originally zoonotic, although some cases of transmission of *L. donovani* from human to human are also known. The different epidemiological cycles are (i) a primitive or sylvatic cycle (human infection is accidental, transmission occurring in wild foci), for example, *L. braziliensis*; (ii) a secondary or peridomestic cycle (the reservoir is a peridomestic or domestic animal, the parasite being transmitted to humans by anthropophilic sand flies), for example, *L. infantum*; (iii) a tertiary, strictly anthroponotic cycle (in which the animal reservoir has disappeared or has not yet been identified, and the sand fly vectors are totally anthroponotic), for example, *L. donovani*. Nevertheless, many unknown factors remain. For example, the main animal reservoir of *L. braziliensis* is still unknown [15]. *L. tropica* was considered to be a strict anthroponosis, but several cases of canine infection have been described [16–18].

Leishmaniasis is a complex disease caused by haemoflagellate obligate intracellular protozoa belonging to the genus *Leishmania*, family Trypanosomatidae of the order Kinetoplastida. Visceral leishmaniasis (VL), cutaneous leishmaniasis (CL), and rarer manifestations such as mucosal leishmaniasis and post-kala-azar dermal leishmaniasis (PKDL) are the major forms of this disease. Parasites are transmitted by female sandflies via anthroponotic or zoonotic cycles [19]. Theparasites have a dimorphic lifecycle; flagellated promastigotes develop in the gut of female Phlebotomine sandflies to infectious forms that are transmitted to mammalian hosts [20, 21]. Inside the host, the parasites survive and multiply as amastigotes within parasitophorous vacuoles (PVs) of macrophages [22, 23]. 

Depending on the transmission cycle, VL and CL are considered to be either anthroponotic (AVL/ACL) or zoonotic (ZVL/ZCL). Anthroponotic VL is caused by *Leishmania donovani* and is mainly distributed in the Indian subcontinent where it accounts for 70% of the burden of VL with estimated annual incidence of 500,000 and 50,000 deaths each year [24], a death toll that is surpassed among the parasitic diseases only by malaria.

**Figure 1:**
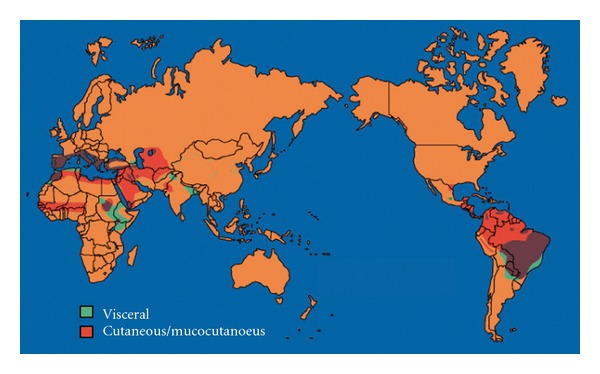
Geographical distribution of leishmaniasis worldwide [37].

Both figures are approximations as VL is frequently not recognized or not reported [25, 26]. The majority (>90%) of cases occur in just six countries—Bangladesh, India, Nepal, Sudan, Ethiopia, and Brazil (Figure 1). Severe VL epidemics have been reported in the past in southern Sudan; in context of civil war and famine, VL killed an estimated 100,000 people out of a population of 280,000 between 1984 and 1998 [27]. As India, Nepal, and Bangladesh harbor an estimated 67% of the global VL disease burden [28], the commitment of the government of these countries to launch regional VL elimination programme is welcome. The target of this programme is to eliminate VL as a public health problem by 2015, by using a local approach to reduce the annual incidence of VL to less than one case per ten thousand individuals. Visceral leishmaniasis (VL) results in death if not treated, the majority of leishmaniasis deaths go unrecognized, and even with treatment access, VL may result in case-fatality rates of 10–20% [29–31]. In East Africa, it causes around 50,000 annual cases, in the form of epidemic outbreaks distributed in scattered displaced populations with a high death rate. Post-kala-azar dermal leishmaniasis (PKDL), which is developed in 5–50% of AVL patients depending on geographical areas, requires lengthy and costly treatment with a low efficacy [32, 33]. The most relevant factors behind the spread of AVL are the increasing transmission in urban areas with large numbers of immigrants living in poor conditions, the breakdown of social and health structures, malnutrition inducing weakening of the immune system, and finally HIV-*Leishmania* coinfection. The HIV-VL coinfection is characterized by frequent relapses and a high fatality rate, and cases are considered to constitute an important infectious reservoir [34]. Zoonotic VL is caused by* L. infantum *and is widely distributed in Central Asia, Middle East, the Mediterranean, and Brazil. Up to 50,000 annual cases may be caused by this form worldwide, with a scattered distribution. 

CL is commonly known as oriental sore. Its causative agents are *Leishmania major*, *L. tropica*, *L. aethiopica*, and *L infantum* in old world and *L. mexicana*, *L. venezuelensis*, *L. amazonensis*, *L. braziliensis*, *L. panamensis*, *L. guyanensis*, *L. peruviana,* and *L. chagasi* in new world. It produces skin lesions mainly on the face, arms, and legs. It is frequently self-healing in the old world but when the lesions are multiple and disabling with disfiguring scars, it creates a lifelong aesthetic stigma. In Central Asia, the Middle East, North Africa, and some sub-Saharan countries, ZCL caused by *L. major* accounts for 500,000 cases every year. Outbreaks are typical in rural areas and depend on fluctuations in the rodent population. ACL caused by *L. tropica* is transmitted in urban zones and affects around 400,000 patients annually. Massive outbreaks have occurred in overcrowded suburbs with poor housing and deteriorated environmental conditions [35]. ZCL caused by *L. aethiopica* is present in Ethiopia and is the most neglected form of CL despite 50,000 annual cases and a potential serious clinical progression, including diffuse CL and to a lesser degree mucocutaneous leishmaniasis. In South America, around 300,000 new cases of ZCL occur annually. *Leishmania braziliensis* is responsible for nearly 90% of all CL cases. Species belonging to the subgenus *Viannia* (*L. braziliensis*, *L. panamensis, L. peruviana,* and *L. guyanensis*) are capable of causing mucocutaneous leishmaniasis. No or incomplete treatment of CL is associated with the subsequent development of mucocutaneous leishmaniasis. It is estimated that there are 4000 new mucocutaneous leishmaniasis cases every year [36]. The subgenus *Leishmania *groups comprise two main species, *L. mexicana*, causing a form of CL that heals spontaneously but can sometimes cause necrosis of the external ear (the “chiclero” ulcer), and *L. amazonensis*, which can in some cases manifest as diffuse CL in patients with weak immune systems.

Although leishmaniasis affect 98 countries in the world, it should be stressed that 90% of VL cases occur in India, Bangladesh, Sudan, Brazil, Nepal, and Ethiopia, and 90% of CL cases occur in Afghanistan, Algeria, Ethiopia, Sudan, Iran, Iraq, Saudi Arabia, Syria, Brazil, and Peru [6]. Apart from VL and CL, diffused cutaneous leishmaniasis is also a type which is difficult to treat due to disseminated lesions that resemble leprosy and do not heal spontaneously. This form is especially related to a defective immune system and it is often characterized by relapses after treatment. 

Mucocutanoeus leishmaniasis is also called “espundia” in South America. Causative agents of MCL in old world are *Leishmania aethiopica* (rare) *L. major* and in new world are *L. mexicana*, *L. amazonensis*, *L. braziliensis*, *L. guyanensis,* and *L. panamensis*. The parasite invades the mucocutaneous region of the body and spreads to the oronasal/pharyngeal mucosa. The soft tissues and cartilage of the oronasal/pharyngeal cavity undergo progressive erosion. In contrast to cutaneous leishmaniasis, these lesions do not heal spontaneously. Suffering and mutilation are severe, and death occurs as a result of bronchopneumonia or malnutrition. There is always a large danger of bacteria infecting the already open sores. Reconstructive surgery of deformities is an important part of therapy [36].

The classification of *Leishmania* was initially based on ecobiological criteria such as vectors, geographical distribution, tropism, antigenic properties, and clinical manifestations [38–41]. However, biochemical and molecular analysis showed that pathological and geographical criteria were often inadequate and thus, other criteria such as the patterns of polymorphism exhibited by kinetoplastid DNA (k-DNA) markers, proteins, or antigens came to be used to classify* Leishmania* [42–49].

The genus *Leishmania* comes under subkingdom Protozoa, order Kinetoplastida, and family Trypanosomatidae. Initially, species classification was based on various extrinsic criteria such as clinical, geographical, and biological characteristics, for example, *L. guyanensis *(isolated in Guyana), *L. peruviana* (isolated in Peru), *L. infantum* (isolated from a child in Tunisia), and *L. gerbilli* (isolated from gerbils). Since the 1970s, intrinsic criteria such as immunological, biochemical, and genetic data have been used to define species of *Leishmania. *Use of these molecular techniques led to the publication of a taxonomic scheme by the World Health Organization [50]. New methods of detection, isolation, and genetic identification resulted in a massive increase in the number of species are described. Today, 30 species are known and approximately 20 are pathogenic for humans. These species generally present different epidemiological and clinical characteristics related to different genetic and phenotypic profiles. The validity of the classification scheme, considered by some workers as too arbitrary, has been questioned several times. Debate has centered on *L. panamensis*, *L. peruviana*, *L. chagasi*, *L. infantum*, *L. archibaldi*, *L. garnhami*, *L. pifanoi*, *L. venezuelensis,* and *L. forattinii* [15, 51, 52]. Different studies have already clarified the status of some of these species; for example, *L. chagasi* is accepted as a synonym of *L. infantum* [51] and *L. peruviana* has been validated as an independent species [53].

Very recently, Fraga and his team [54] used sequences of the highly conserved 70-kDa heat shock protein (hsp70 gene) to analyze isolates and strains of different geographic origin, showing that only eight monophyletic groups were detectable against the 17 examined (Table 1).

## 2. Diagnosis 

The reason behind the diagnostic challenges for leishmaniasis is the wide spectrum of clinical manifestations that they present: ulcerative skin lesions developing at the site of the sand fly bite (localized cutaneous leishmaniasis); multiple nonulcerative nodules (diffuse cutaneous leishmaniasis); destructive mucosal inflammation (mucosal leishmaniasis (ML)); disseminated, potentially fatal, and visceral infection (visceral leishmaniasis (VL)) [55]. However, differential diagnosis is important because diseases of other etiologies with a clinical spectrum similar to that of the leishmaniases (e.g., leprosy, skin cancers, and tuberculosis for CL and malaria and schistosomiasis for VL) are often present in areas of endemicity. These main manifestations may themselves deviate, complicating definitive clinical diagnosis even further. Cutaneous leishmaniasis (CL) lesions, for example, may vary in severity (e.g., in lesion size), clinical appearance (e.g., open ulcer versus flat plaques versus wart-like lesions), and duration (e.g., in time of evolution or in time to spontaneous cure). Patient management, screening of asymptomatic infections, surveillance including verification of elimination, and epidemiological studies are some of the areas where diagnostic tests play a major role. 

In all cases, it is desirable to have the diagnosis of leishmaniasis confirmed by the finding of the etiological agent or its antigen or molecule in the sample obtained from the lesion. When these approaches fail, immunological tests are used to provide indirect parameters for the diagnosis.

Ideally, a test should make the distinction between acute disease and asymptomatic infection, as most of the antileishmanial drugs are toxic. Moreover, such tests should be highly sensitive and specific, simple and affordable, but unfortunately some commonly used serological tests like DAT carry some significant drawbacks: the inability to differentiate between clinically active and asymptomatic infections and showing positive long after cure. Molecular diagnostic tools like PCR and real-time PCR are quite sensitive and specific but are cumbersome to perform and have a high cost. DNA-based tests are available in strip formats but these cannot be used in the field [56]. Compared to other diagnostic techniques available, the molecular approaches remain expensive and require technological expertise, and efforts should be made to make PCR platforms more user-friendly and cost-effective, especially in remote areas where leishmaniasis is endemic. 

### 2.1. Parasitological Methods

Demonstration of the amastigote form of the parasite by light microscopic examination of tissue aspirates from spleen, bone marrow, or lymph nodes is regarded as the most suitable diagnostic instrument. In preparations stained with Giemsa or Leishman stain, the cytoplasm appears to be pale blue, with a relatively large nucleus that stains with red. In the same plane as the nucleus, but at a right angle to it, is a deep red or violet rod-like body called a kinetoplast. The sensitivity of the direct examination is low, in case of cutaneous and mucocutaneous leishmaniasis, with a range of approximately 15–70% in the old and the new world [57, 58]. In case of visceral leishmaniasis, the specificity of this technique is high, although the sensitivity varies depending on the tissue used, being higher for spleen (93–99%) than for bone marrow (53–86%) or lymph node (53–65%) aspirates [59]. However, the procedure for splenic aspiration is risky for fatal internal bleeding, so the results are totally dependent on technical expertise and quality of prepared slides/reagents, both of which are often not available in field settings [60].

The microculture method (MCM) is a good method for the diagnosis of visceral leishmaniasis (VL) with samples from both the bone marrow (BM) and peripheral blood (PB). The MCM is superior to the traditional culture method (TCM) as determined by its higher sensitivity in the detection of promastigotes and the more rapid time for emergence of promastigotes. The sensitivity of MCM (100% in BMs and 77.8–100% in PB) is considerably higher than that of the TCM (37.5–100% in BMs and 0–100% in PB) according to decreasing parasite density (*P* < 0.05) [61].

### 2.2. Serological Methods

The serological diagnosis is based on the presence of specific humoral response for which identification of antibodies in the sera of patients is done. Serum-based direct agglutination tests (DATs) using lyophilized promastigotes or urine-based latex agglutination tests (LATs) are used for determining antileishmanial antibodies or antigens in leishmaniasis patients. DAT has a high sensitivity (90–100%) and specificity (95–100%) [62, 63]. However, the major disadvantage of DAT is the need of multiple pipetting, relatively long incubation time, high cost of antigen, and limited production facility of quality controlled antigen. As with any antibody-based test, DAT remains positive for a long time after the disease is cured, thus cannot be used as a test of cure or for diagnosis of relapses [64].

Detecting antigen directly is an excellent method of diagnosing an infection and is more specific than antibody-based immunodiagnostic tests. Agglutination test to detect the antigen has been evaluated extensively in clinical trials, using urine collected from well-defined cases and controls from endemic and nonendemic regions. This test showed 79.1–94.1% specificity and sensitivity of 60.4–71.6% in India [65]. 

Several surface antigens, ribosomal proteins, nuclear proteins, histones, and kinesin-related proteins elicit specific humoral immune responses in VL patients. Recombinant antigens have considerably improved the sensitivity and specificity of immunological diagnosis over crude/total antigens. They are more preferred than other antigens both in immunoblotting as well as ELISA. 

Immunochromatographic strips using K39 antigen have been a quite promising method that has been tested widely. K39 (recombinant) antigen contains 39 amino acids encoded in the highly conserved kinesin region of *L. chagasi*. Using its recombinant product, an immunochromatographic-based strip test is used in which rK39 is fixed on a nitrocellulose paper, and colloidal gold-protein A is used for detection. In the initial clinical evaluation, 100% sensitivity and 98% specificity was observed. ICT suffers from the same disadvantage as DAT, being positive in a significant proportion of healthy individuals in endemic regions and for long periods after cure of VL. 

ELISA has also been used in the serodiagnosis of all types of leishmaniasis but the sensitivity and specificity of ELISA depends upon the antigen used. Most promising results are shown by antigen RK39 with sensitivity and specificity of 100% and 96%, respectively. The antibody titres to this antigen directly correlate with active disease and have potential in monitoring the chemotherapy and in predicting the clinical relapse [66]. Due to the requirement of skilled personnel, sophisticated equipment, and electricity, ELISA is not used in the endemic regions for the diagnosis of VL [67].

### 2.3. Molecular Methods

PCR-based assays form the mainstay of molecular diagnosis of leishmanial infection as they enhance sensitivity, reliability, and rapidity for the benefit of researchers and health professionals. The primers target several multicopy sequences for the diagnosis of human and canine VL which include ribosomal RNA genes [68]; kinetoplast DNA (kDNA) [69]; miniexon-derived RNA (med RNA) genes and genomic repeats [70], the *β*-tubulin gene region [71], glycoprotein 63 (gp63) gene locus [72], and internal transcribed spacer (ITS) regions [73].

ThePCR-ELISAhas shown promising results for diagnosing visceral leishmaniasis (VL) in blood samples. However, the method has been validated mostly with HIV-positive patients who are known to have high levels of parasitaemia. As far as blood samples are concerned,PCR-ELISAis more sensitive (83.9%) than conventional PCR (73.2%) and demonstrated 100% and 87.2% specificity when healthy controls who had never travelled to a VL-endemic area and controls from a VL-endemic area as references, respectively, were used [74].

RT-PCR simply refers to amplification of DNA (by PCR) that is monitored while the amplification is occurring. The benefit of this RT-PCR is that it allows the researcher to better determine the amount of starting DNA in the sample before the amplification by PCR. Also real-time PCR can distinguish specific sequences from a complex mixture of DNA. However, its application requires the availability of primers and probes that must be selected according to very rigid conditions, which cannot always be easily applied [75].  Table 2 depicts the properties of few generally used diagnostic tools for leishmaniasis and PKDL. 

### 2.4. Developments in Antileishmanial Drugs

Pentavalent antimonials were developed in 1945; the generic sodium stibogluconate (pentosan) and branded meglumine antimoniate, and so forth, are the first choice in the treatment of both visceral and cutaneous leishmaniases over more than five decades where resistance is not reported [91]. To overcome the challenge of clinical resistance to antimony, pentamidine has been tried for the treatment of visceral leishmaniasis and was the first drug to be used for patients refractory to pentavalent antimony (SbV) [92]. This drug is associated with serious adverse events like insulin-dependent diabetes mellitus, shock, and hypoglycaemia and death in significant proportion of patients. The declining efficacy, resistance, and serious toxicity associated with the drugs have made it unsuitable as a viable alternative to SbV for kala-azar patients [93, 94]. Amphotericin B is one of the most effective antileishmanial drug, which induces high cure rates. Use of formulation of amphotericin B, a polyene antifungal drug for treatment of leishmaniasis is biochemically rational because the target of the drug is ergosterol, which is the major membrane sterol of *Leishmania *species. Due to high affinity of amphotericin B for sterols, aqueous pores are formed in the membrane leading to increased membrane permeability and killing of *Leishmania *[95]. The need to develop less toxic, more effective formulation of amphotericin B has led to new clinical formulation of amphotericin B in which deoxycholate has been replaced by other lipids. Miltefosine, an alkyl phospholipids developed as an antitumor agent, has an excellent antileishmanial activity.Paromomycin is an aminoglycoside antibiotic with unique antileishmanials activity. Paromomycin is currently in phase IV clinical trials against leishmaniasis [96]. Sitamaquine, a primaquine analogue (8-aminoquinolene), is another orally administrable compound (Table 3). To date, little is known about its efficacy and toxicity. However, there is a need to go for combitorial chemotherapies which are under process and provide better outputs. 

## 3. New Potential Drug Targets

The irony of the disease leishmaniasis is that it is the only tropical disease, which is being treated by nonleishmanial drugs [97]. Much of the focus till recent times was being made only on drug trials/combination therapy of available nonleishmanial drugs, evaluation of diagnostic and prognostic capability of available tools, and very little emphasis was being paid on other aspects by leishmanial biologists and researchers. Recently much emphasis has been given on novel control strategies in terms of new drug targets and vaccine candidates. 

The route to drug target identification has been through comparative biochemistry of host and parasite enzymes, metabolites or protein identified in parasite. Biochemical analysis, genome sequencing of three *Leishmania* species (*L. major, L. braziliensis, *and* L. infantum*) has identified potentially useful target enzymes, transporters, metabolites, and hypothetical proteins that are distinct to parasite and their mammalian host. The genome mining will also aid in large scale proteomics studies generating expression profiles of *Leishmania* parasites and gene targets for treatment development. Search of new potential drug targets mainly focuses on biochemical and metabolic pathways essential for parasite survival. The target enzymes of these pathways should have significant structural and functional differences from their mammalian counterparts for selective inhibition of target sites. Further, strategies to target more than one enzyme of a metabolic pathway simultaneously may prove more usefulness and effectiveness. Biological studies for the function of 50% of *Leishmania* genes are lacking. The comparative genome study would provide a route to find those that might be essential to each species [98].

## 4. Thiol Metabolism

The enzymes of thiol metabolism/thiols, of parasitic protozoa, are different from those of mammals in many ways. *Trypanosoma* and *Leishmania* are most remarkable in that they have trypanothione reductase (TR) instead of glutathione reductase (GR). This enzyme is responsible for maintaining the parasites, reducing intracellular milieu by keeping trypanothione [*N* ^1^,* N8*-*bis-*(glutathionyl) spermidine] in the dithiol state. The crucial role of TR for thiol homeostasis and its absence from mammalian cells suggest that it might be well suited as a target molecule for rational drug development. The trypanothione system, which replaces the nearly ubiquitous glutathione/glutathione reductase (GR) system, protects the parasites from oxidative damage and toxic heavy metals and delivers the reducing equivalents for DNA synthesis. The parasite system is far less efficient than mammalian glutathione peroxidases in detoxifying hydroperoxide, but has the advantage of much broader substrate specificity, with lipid hydroperoxides also being reduced. The relatively low activity of the tryparedoxin peroxidase system is in accordance with the high sensitivity of the parasites to oxidative stress. Trypanosomes and *Leishmania* have superoxide dismutase (SOD), but lack catalase and glutathione peroxidase. Thus, the trypanothione system seems to be the only mechanism to detoxify hydrogen peroxides.

Trypanothione is kept reduced by the flavoenzyme TR. Several reverse genetic approaches have undoubtedly shown that TR is essential in different *Leishmania* species as well as in bloodstream of *T. brucei* [99] and is thus an attractive target molecule for structure-based drug design. Castro-Pinto et al. [100] have reported the cloning, sequencing, and expression of the TR encoding gene from *L. (L.) amazonensis*. A 3D homology model for L. amazonensis TR was constructed based on the previously reported *Crithidia fasciculata* structure. Within the past 15 years, numerous compounds have been elucidated that inhibit TR, but not human GR, which is the closest related host enzyme. Despite knowledge of the three-dimensional structure of the protein and of complexes with its substrates and an inhibitor, as well as several high-throughput and virtual screening approaches, inhibitors of TR that are suitable to enter the clinical phase are still elusive. This lack of success might be attributable to several factors. The extremely wide active site of the parasite enzyme represents an obstacle for a structure-based drug design. In addition, the pharmacokinetic properties of the potential inhibitors are crucial because insufficient uptake, rapid extrusion, or metabolism plays significant roles in determining the *in vivo* efficacy of a drug. Recently, the biological activities of a series of mesoionic 1,3,4-thiadiazolium-2-aminide derivatives were evaluated to determine their effect on trypanothione reductase (TryR) activity in *Leishmania* sp. and *Trypanosoma* cruzi. MI-4-NO(2) showed enzyme inhibition effect on extracts from different cultures of parasites, which was confirmed using the recombinant enzyme from *T*. *cruzi* (TcTryR) and *Leishmania infantum* (LiTryR) [101]

Thioredoxin reductase (TrxR) is a pyridine nucleotide-disulphide oxidoreductase as GR, TR, and lipoamide dehydrogenase. TrxR maintains the levels of reduced thioredoxin, a protein involved in the activity of ribonucleotide reductase, transcription factors, and cell signaling and the detoxification of reactive oxygen species. Most studies to date on TrxR of parasitic protozoa have concerned the enzyme of *P. falciparum*. Current evidence suggests that it is a promising drug target, although validation is awaited. The absence of this pathway in mammalian host and trypanosomatids sensitivity towards oxidative stress, trypanothione reductase, and enzymes of trypanothione metabolism is an attractive drug target for antileishmanial drug designing [102]. Homology modeling of *Leishmania infantum*, TR, and mammalian glutathione reductase shows a remarkable difference in their three-dimensional and catalytic active sites. Hence, specific inhibitors designed against TR may be an ideal drug that will stop parasite growth without altering host glutathione reductase (GR) activity.

### 4.1. Sterol Biosynthetic Pathway

The enzymes of this pathway are attractive targets for the specific treatment of leishmaniasis, because the etiological agents for the disease, that is, the leishmanial parasites have a strict requirement for specific endogenous sterols (ergosterol and analogs) for survival and growth and cannot use the abundant supply of cholesterol present in their mammalian host. There are differences in the enzymes in the biosynthetic pathways of ergosterol and cholesterol. A number of enzymes in the ergosterol biosynthetic pathway have been investigated as potential drug targets for these organisms and have shown great promise. Thus, C14-demethylase, sterol 24-methyltransferase, 3-hydroxy-3-methylglutaryl CoA reductase, squalene epoxide, squalene synthase, and farnesyl pyrophosphate synthase have been studied both individually and in combination, with varying degrees of success [103, 104]. Ergosterol biosynthesis inhibitors with potent *in vitro* activity and special pharmacokinetic properties in mammals can induce radical parasitological cure in animal models of several forms of leishmaniasis [105].

Trypanosomatids contain predominantly ergostane-based sterols, which differ from cholesterol, the main sterol in mammalian cells, in the presence of a methyl group in the 24 position. The methylation is initiated by S-adenosyl-L-methionine: Delta (24 (25))-sterol methenyltransferase, an enzyme present in protozoa, but absent in mammals. The importance of this enzyme is underscored by its potential as a drug target in the treatment of the leishmaniasis [106]. The C-24 transmethylation reactions involving S-adenosyl-L-methionine as the methyl donor and a Δ^24(25)^-sterol or Δ^24(24′)^-sterol substrate can be inhibited by various azasterols with a nitrogen substitution in the side chain and such compounds have been tested against trypanosomatids [107].

### 4.2. Polyamine Biosynthetic Pathway

The polyamine pathway of protozoan parasites has been successfully targeted in antiparasitic therapies. Polyamines are ubiquitous organic cations found in all eukaryotic cells and play critical role in key cellular processes such as growth, differentiation, and macromolecular biosynthesis. During the course of inhibition of any of the polyamine, the parasite cannot synthesize trypanothione, a conjugate of spermidine and glutathione that is unique to *Trypanosoma* and *Leishmania*. Trypanothione is a reducing agent with many protective and regulatory functions and consequently its depletion proves detrimental to the parasites. Recent studies on polyamine supplementation show that *L. donovani* lacks an intact back conversion pathway, thus the pathways operating in promastigote stage of parasite differ crucially from that in the host. Inhibitors of polyamine biosynthetic pathway have shown antileishmanial activity. Adomet DC inhibitor cures animal leishmanises but has not been tested on humans and seeks further experimental studies [108]. Arginase provides a building block for production of polyamines so it has been touted as a potential antileishmanial drug target, because N(omega)-hydroxyarginine, an inhibitor of arginase that is produced by the macrophages during the formation of nitric oxide, can reduce polyamine levels in *Leishmania* amastigotes and lowers parasitic loads [109, 110]. Other enzymes of this pathway which are under study as antileishmanial target are ornithine decarboxylases, S-adenosylmethionione, and Spermidine synthase.

### 4.3. Glycosomal Machinery

In all kinetoplastida studied so far the majority of the glycolytic enzymes are localized in organelles called glycosomes, whereas in other organisms these are cytosolic. As a result of this compartmentation, many regulatory mechanisms operating in other cell types cannot work in trypanosomes as reflected by the insensitivity of the glycosomal hexokinase (HK) and phosphofructokinase (PFK) to compounds that act as activity regulators in other cell types [111, 112]. Blocking of parasite enzyme without producing damage to glycolysis in host remains challenging. Several approaches have been considered—(1) exploitation of metabolic differences; (2) exploitation of differences in 3D structure; (3) exploitation of unique reactive residue in or near the active site of the parasite enzyme

*Leishmania* like other trypanosomatids depends solely on its host for carbon source to fulfill its energy requirements. The amastigotes uptake blood glucose from mammalian blood stream and receive other essential components like fatty acids, amino acids from phagolysosome of macrophages. Due to the result of the metabolic activities of glycosomes, superoxide radicals are generated as side products in large amount. To protect glycosomal enzymes from superoxide radical toxicity, Fe-superoxide dismutases (FeSods) are evolved in *Leishmania *species. More importantly, FeSod is absent in mammalian counterpart, so it could be used as effective drug target.

### 4.4. Cyclin-Dependent Kinases

Cyclin-dependent kinases (cdks) play crucial role in cell division cycle, transcription, apoptosis, and differentiation. In *Leishmania mexicana*, disruption of cdk-related kinase 3 (CRK3) leads to change ploidy level of the cell, though it was avoided when extra copy of CRK3 was expressed from episome, ensuring that CRK3 is essential [113]. The chemical inhibitors of class indirubin of CRK3 hampers the parasite viability within macrophage, proving the validity of CRK3 as potential drug target [114]. In *Leishmania donovani*, it was recently shown that glycogen synthase kinase (LdGSK3) is also involved in cell cycle control and apoptosis as validations based on indirubin test [115], exploiting the LdGSK3 as potential drug target in combination with CRK3. Likewise, other cdk may also be explored as possible targets.

### 4.5. Folate Metabolism

Dihydrofolate reductase (DHFR) is a key enzyme in folate metabolism, linked to the production of thymidine. DHFR reduces dihydrofolate to tetrahydrofolate using NADPH as cofactor. Therefore, inhibition of DHFR prevents biosynthesis of thymidine and as a consequence, DNA biosynthesis. Fortunately, this enzyme from *Leishmania major* and *Trypanosoma cruzi* has been crystallized and the structural data can be used to exploit structural difference between parasite and human enzymes that may help to design selective DHFR inhibitors [116, 117]. An approach to discover novel parasite DHFR inhibitors using database mining has also been made to search the Cambridge structural database but DHFR as drug target requires more attention [118, 119]. In addition, it has been shown that the enzyme dihydrofolate reductase-thymidylate (DHFR-TS) that catalyzes conversion of dihydrofolate from methyhylene tetrahydrofolate (M-THF) and thymidine, which is related to parasite survival and the parasite lacking this enzyme is not able to survive in animals [120].

## 5. Mitochondria

The unique mitochondrial features of *Leishmania* make this organelle an ideal drug target while minimizing toxicity.*Leishmania* has a single large mitochondrion which is distributed in branches under the subpelicullar microtubes and a specialized region rich in DNA called the kinetoplast. Fonseca-Silva et al. demonstrated that the effect of the quercetin is associated with ROS production leading to mitochondrial dysfunction, ultimately causing parasite death [121]. The effects of several drugs that interfere directly with mitochondrial physiology in parasites such as *Leishmania* are under study. The unique mitochondrial features of *Leishmania* make this organelle an ideal drug target while minimizing toxicity.

## 6. Conclusions

All the neglected tropical diseases together take the toll of millions of lives every year. Leishmaniasis is a fatal disease which affects the people living below poverty line. The recent years have witnessed extraordinary progress in diagnosing and treating *Leishmania* infection. Lack of efficiency, high price, and growing resistance of the current antileishmanials imply the importance of search for new targets to be focused for making drugs against *Leishmania.* The challenge is to convert such studies in effective strategic programmes aimed to control and eradicate the disease. 

## Figures and Tables

**Table 1:** Nomenclature of *Leishmania* species based on heat shock protein 70 (hsp 70) gene sequences [54].

Name of the organism	Geographical distribution
*L. donovani *	China, Indian subcontinent, Ethiopia, Sudan, Kenya, Iran, Saudi Arabia, and Yemen
*L. infantum/L. Chagasi* As given [51]	Albania, Algeria, France, Greece, Italy, Morocco, Portugal, Spain, Syria, Tunisia, Turkey, Yemen, Argentina, Bolivia, Brazil, Colombia, Ecuador, El Salvador, Guadalupe, Guatemala, Honduras, Martinique, Mexico, Nicaragua, Paraguay, Suriname, and Venezuela
*L. archibaldi *	India, Sudan, Ethiopia, Lebanon, and Israel
*L. tropica *	Afghanistan, Algeria, Azerbaijan, Greece, Iran, Iraq, Israel, Morocco, Tunisia, Turkey, and Yemen
*L. aethiopica *	Ethiopia, Kenya
*L. major *	Afghanistan, Algeria, Chad, Iran, Iraq, Israel, Libya, Mauritania, Morocco, Syria, and Sudan
*L. mexicana *	Belize, Colombia, Costa Rica, Dominican Republic, Ecuador, Guatemala, Honduras, Mexico, Panama, and Venezuela
*L. amazonensis *	Bolivia, Brazil, Colombia, Costa Rica, Ecuador, French Guyana, Panama, Peru, and Venezuela
*L. garnhami *	Venezuela
*L. guyanensis *	Brazil, Colombia, Ecuador, French Guyana, Peru, Suriname, and Venezuela
*L. panamensis *	Belize, Colombia, Costa Rica, Ecuador, Honduras, Nicaragua, Panama, and Venezuela
*L. naiffi *	Brazil, French Guyana, Ecuador, and Peru
*L. braziliansis *	Argentina, Belize, Bolivia, Brazil, Colombia, Costa Rica, Ecuador, Guatemala, Honduras, and Nicaragua
*L. peruviana *	Peru

**Table 2:** Diagnostic methods for leishmaniasis and PKDL.

Diagnostic methods	Test name	Clinical specimen	Sensitivity	References
Parasitic detection methods	LD bodies	Lymph node, bone marrow, splenic, and slit aspirates	spleen (93–99%) than for bone marrow (53–86%) or lymph node (53–65%) aspirates	[59, 76]
	Culture (MCM)	Blood, lymph node, bone marrow, splenic, and slit aspirates	100% in bone marrow and 77.8–100% in peripheral blood	[77]
	Culture (TCM)	Blood, lymph node, bone marrow, splenic, and slit aspirates	37.5–100% in bone marrow and 0–100% in peripheral blood	[77]

Serological methods	DAT	Serum, urine	94.8%	[78–81]
	ELISA/immunoblotting soluble antigens	Serum, urine	100%	[82–84]
	Recombinant antigens (rK39, rK26, rHSP70)	Blood, serum, and antigens	100%	[85–88]

Molecular methods	PCR	Blood, serum, urine, lymph node, bone marrow,splenic, and slit aspirates	73.2%	[89, 90]
	PCR-ELISA	Blood, serum	83.9%	[90]
	Real-time PCR	Lymph node, bone marrow, splenic, and slit aspirates	90–100%	[75]

**Table 3:** Current scenario of available chemotherapy drugs.

Drug	Properties and administration	Comments	Reactions in patients with CL/VL
Pentavalent antimonials	Polymeric organometallic complexes, intravenous, or intramuscular	For VL and CL. Drug resistance in Bihar, India. Variable response in different forms of CL. Generic sodium stibogluconate (SSG) has made treatment cheaper	Pain, erythema, oedema, abdominal pain, nausea, and thrombocytopenia

Amphotericin B fungizone	Polyene antibiotic, Fermentation product of Streptomyces nodus, intravenous	For VL, CL, and complex forms of CL, for example, MCL. first-line drug for VL in India where there is antimonial resistance	Infusion related, azotemia, anemia, or hypokalemia

Amphotericin B ambisome	Unilamellar liposome, intravenous	Most effective lipid formulation for VL and available at $18/50 mg ampoule via WHO used for complex forms, such as PKDL and MCL	Hypotension,anorexia,nausea,vomiting, and headache generalized weakness.

Miltefosine	Hexadecylphosphocholine, oral	First oral drug for VL. Effective against some forms of CL contraindicated in pregnancy	Nausea, vomiting and/or diarrhea, raised creatinine, and raised LFT's

Amphotericin B formulations	Lipidic formulations, intravenous	Other lipid formulations, including Abelcet, Amphocil, Amphomul, and multilamellar liposomes have been in clinical studies, mainly for VL	Shaking chills, nausea, hypotension,anorexia,headache, and vomiting,

Paromomycin	Aminoglycoside (also known as aminosidine or monomycin), fermentation product of *Streptomyces rimosus*. Supplied as sulphate. Intramuscular for VL and topical for CL	Registered for VL in India, completed Phase III trials for VL in East Africa where less effective in Sudan. Topical formulation (12%) with methyl benzethonium chloride available for CL. Topical with gentamicin and surfactants in Phase III trial	Pain, erythema, oedema, blisters, and ototoxicity

Pentamidine	Diamidine, as isethionate salt, intramuscular	For specific forms of CL in South America only	Nausea, vomiting, diarrhea, hyperglycemia, and cardiotoxicity

Sitamaquine	8-aminoquinoline analog, orally active	Tested in VL patients in Kenya and Brazil with limited success	Abdominal pain, headache, vomiting, dyspepsia, and cyanosis
